# About Livers

**Published:** 1881-01

**Authors:** Ben. H. Pratt


THE BISTOURY.

Vol. XVI. ELMIRA, N. Y., JANUARY, 1881. No. 4.

### Contributions.

### ABOUT LIVERS.

BEN. H. PRATT.

Everybody, alas! has a liver. Time was when people didn't know that they had any liver, to any great extent. O, the bliss of that delightful ignorance! Alack and well-a-day that we should ever have been lifted out of that exquisite ignorance into our present miserable knowledge! And we call this an advanced age, and are said to view matters of science and art from a loftier plane than the world ever before stood upon! We are supposed to stand squarely upon this modern elevation of our civilization and pity the benighted ones of former ages, because they knew so little of many things of which we know so much. Among things known in the age referred to were not livers. Happy ignorance! Think of the blissfulness of an absolute unconsciousness of having a liver! We don't know who discovered the liver. Wish we did. Hope no monument rises over his dust. But history does not give us the displeasure of an acquaintance even with his mythhood, so we cannot curse his memory as it would give us pleasure to do. Yet maledictions would do him no harm, probably, according to accepted psychic theories. Still it would be a comfort to relieve one's self of a few conventional phrases, not laid down by orthodox theological authorities, by distributing them around among the particles of his dust. Regrets, however, are useless, as the mischief has been done—the human liver has been discovered, and the science of medicine has advanced upon this discovery with gigantic strides. That organ—they say it's an organ—has done more toward building up the practice of medicine and palatial residences for practitioners thereof, than any other ten distinct component parts of the human anatomy. There is no means of computing the debt that society owes to the liver and to those who have meddled with it in the way of alleged treatment. Perhaps the doctors' ledgers, viewed on the debit side, might convey a faint idea of this debt. They who have been called upon to administer the estates of deceased physicians that passed to their final rest after years and years of an immense practice, may entertain a mere inkling of the enormousness of this obligation ; but the tithe thereof will never be computed because it is incomputable by any known numerical system. Since the discovery of the organ referred to, the number of diseases that have been invented by medical and diabolical genius, and each of which is directly consequent upon the consciousness of the existence of a liver, is beyond computation. The mere reflection upon the length of a list of diseases directly traceable thereto, is bewildering ; but when one begins to ponder upon the legion of maladies indirectly referable to this unhappy gland—they say it's a gland—one's brain begins to whirligig like a double acting, reversible motion, quintuplex lawn sprinkler under a maximum hydraulic power, and it scatters with a similarity thereto that makes the comparison peculiarly apt. But let us not risk pursuit of this subject in this distracting direction ; let us get back to our livers.

The liver is the largest gland within the human anatomy. The books tell us this. There's no need that they should do so, because, as soon as one is made aware of the fact that he has a liver, he will never thereafter dispute its extent. In fact, one would be much more likely to maintain the assertion that the liver is greater than one's whole system, were it not for the axiomatic truth which confronts him with the certainty that the part cannot be greater than the whole. Even with this truth before one, one is half inclined to make an exception in the case of one's liver. It required no genius to discover the mastodonic characteristic of the liver. Just as soon as one knows he has a liver, he knows he's got the biggist thing in the way of a pest, and an abomination and a misery that man can have. The only surprising circumstance connected with the possession of a liver is, that it is an organ of the tenement we are pleased to call the body—an addendum thereto—and that the body is not an appendage of the liver.

The liver is of a reddish hue. The books tell us this, but very few believe it. Reasoning from the stand-point of comparative anatomy, we cannot well deny the bookish assertion. Any boy that has been sent to market for fifteen cents worth of liver will tell you that liver is red. He is sure of this. But this is dead liver. Still, there are boys who have had curiosity enough to wait at the shop for a liver until one was got ready, and have seen the gland jerked all quivering from the scarcely dead animal of the shambles, and he will tell you that livers are always red. Yet we have heard men called "white livered." There appears to be some disgrace attached to a man whose liver is of this complexion, however, for we have noticed that the prefix "white livered" is always associated with some other objurgatory remark. Hence it is that we regard the alleged lividity of the liver as abnormal, and this strengthens the theory contained in the books to the effect that it is of a reddish cast. This, however, is not an important consideration, and but for our great regard for the verities in treating upon this subject, we should not have been thus particular about the color of the liver.

The liver is said to be round, as to its superior or upper parts, and flat as to its inferior or lower. This corresponds with our knowledge of livers in the lower animals, and we do not propose going into any elaborated argument to prove the truth or fallacy of the statement. It makes little difference whether it be round, flat, ovoid or square, for that matter ; though we must say that we've never known anything square about any liver with which we are, or have been familiar.

The liver is lobed. That is to say, it has several thick leaves, and these leaves flip-flop over each other, against each other, and around each other in a most remarkable fashion. We don't now think of anything to compare a set of liver to unless it be to three or four small elephants' ears, stained with polk-berry juice, stuck together at the roots with mucilage, and then blown up with a goose quill to the desired size. That would pass for a liver before a class of medical students, and none be the wiser. Some doctors we have met would lecture an hour on an artificial liver of this kind and never detect the fraud. In this case the "fraud catch a fraud" doesn't seem to apply.

The liver dwells under the diaphragm—right under it. This may have something to do with its cussedness. The liver, like any other mean thing, can't endure that anything within sight should be above it. The diaphragm, too, has a wavy air about it that is doubtless tantalizing, and if the liver has a tithe of the sentient quality about it, the relative positions of the two would strengthen the theory upon which we account for the excessive meanness of the organ upon which we are wasting so much attention at this time. We touch upon this only hintfully however, and pass on.

Livers nestle, mostly, rather to the right side of the body, but this location is not absolute, nor is it of material consequence. Even people with suicidal or murderous proclivities wouldn't give a cent to know the exact location of the liver. Nobody ever aims at the liver to effect death. We don't know why, but they don't. If some one would only try the experiment, we think one could die from a shattered liver as comfortably as from a collapsed heart. It might be a slower way to pass off, but one would have the satisfaction of getting even with his liver at last, if he could live to know it was all smashed to shreds. This is an age of experiment, and shooting or stabbing one's self through the liver would sound sensational-like in the associated-press paragraph that any one is entitled to when he goes off in a way out of the ordinary.

There is work ascribed to the organ called the liver. Its alleged work is to take the food that is absorbed by or taken into the venous blood, work it over in a way peculiar to itself—swash it around, probably—and effect such a change as to produce what is called bile. Everybody knows bile. What this generation doesn't know about bile is very trifling. The liver's work is to secrete this bile from the venous blood, as we stated. Now, livers are a good deal like folks. Some are much too smart, and some are too infernally lazy to exist. Very few people now-a-days, have easy-going, moderate livers ; that is, livers that work to suit—just work enough—not too much. It is provoking to have people about that are too busy ; it's quite as provoking to have those around you who don't seem to have any business at all. Just so with livers. The smart, active liver, always makes too much bile. The slow, torpid liver, doesn't make enough. One's as bad as 'tother. The man with too much bile is in a dreadful state; so is the man without enough. We'd as soon be one as the other. One is eternally talking about his bile—wondering how he can get rid of it—taking something all the time for it. It gets into his brain, and so affects the molecular structure thereof, that he becomes the prince of credulists—believes everything recommended to him, in the way of remedies, to be good for what ails him, and he takes everything. He becomes a depot for all the drugs the market affords, and all the domestic remedies of a sympathizing community. He loads himself full and then retches himself empty, trying to drive out, scour out, slush out or heave out his bile. The fellow without enough bile, is perpetually taking the same thing the other fellow takes, but with the view of making his liver act—prodding or lashing it into some sort of animation—getting up a general commotion and row inside of him in order to prevail upon the liver to spring to and do something. But it is very seldom that a slow liver can be urged to do anything except on a spurt. It has no momentum, a slow liver hasn't. Calomel will make it fly around and do some splendid work for a few days, but you might as well expect a boy to pick stone right along and not bush, as to hope that your liver will attend to business without being continually punched. Calomel is the liver's grog. So long as you keep up the stimulent, the liver will turn off heaps of work, but you cannot count on it for any length of time. This is the chief defect in livers, and there are more drugs used, and more fortunes swallowed up, and more suicides committed, and bigger medical practices built up on this characteristic of the liver than upon those of all the other organs combined. Every medical advertisement sets forth that the compound advertised is a liver invigorator, and as everybody is conscious of having a liver and ninety-nine out of every hundred are the victims of their livers, people go to experimenting with every nostrum in the market, and before it's discovered to be a humbug, the humbugger has got rich and retired.

The liver is said to have other work to do besides gathering up bile. It makes starch out of the blood, the books say. In this the liver is certainly more successful as an industrial agent of the human economy. At least we rarely hear of a liver's being behind in its starch making. It evidently likes this part of its labors better than it does the bile business. It is unfortunate that the bile making—that is, if we must have bile—has not been delegated to some other organ. There's the spleen, for instance. That seems to be a specially favored organ, as nobody knows what it has to do. It has no visible employment whatever, and it might take to the manufacturing of bile, like it, and make a success of it. It certainly couldn't make a more signal failure in the business than the liver has made. But it doesn't seem to be located on any of the trunk lines that carry the raw material—the blood—and we presume it would be quite inconvenient for it to embark in the enterprise referred to.

The liver has still another kind of work to do. It must take the starch it makes, and from this manufacture sugar. It is asking too much of the liver to do all this. Probably the entire difficulty arises from the fact that the liver has been given too much to do, and as its tastes incline to the starch and sugar making, and it is obliged to neglect one of the three, it lets the bile business drag. It is possible that in anathematizing the liver we may be doing it a gross injustice. Let the reader put himself in the liver's place for a moment, and find himself with bile making, starch-making and sugar-making on his hands. Isn't it reasonable to suppose that if either had to be neglected, the bile business would be the first to suffer? We can't blame the liver for shirking in the direction of the bile. We would, if we were a liver, and had any pride or self-respect about us. Looking at the matter in this light, we begin to feel as though we had, at sundry times and in divers ways, done an injustice—several injustices, in fact,—to the liver. It has doubtless endured more abuse, verbal, mental and druggal, than any other organ of our anatomical economy, and now that we come to seriously think upon the possible excuses or reasons—as hereinbefore set forth—for the alleged lassitudinal phenomena exhibited by the liver, we confess to a few qualms of conscience for what we've said and thought and swallowed with punitive intentions upon our own liver.

We designed, in starting this article, to speak extendely upon the subject of livers, but having scarcely reached our subject as yet, and being conscious that we neither own the BISTOURY, nor have even a quarter's lease of it, we deem it wise to suspend at this point.

